# An evaluation of suspected cases of Hantavirus infection admitted to a tertiary care university hospital in Düzce, Turkey, between 2012 and 2018

**DOI:** 10.3906/sag-1912-123

**Published:** 2021-02-26

**Authors:** Nevin İNCE, Kürşad ÖNEÇ, Tansu SAV, Mehmet Ali SUNGUR, Dilek MENEMENLİOĞLU

**Affiliations:** 1 Department of Infectious Diseases and Clinical Microbiology, Düzce University Faculty of Medicine, Düzce Turkey; 2 Department of Nephrology, Internal Diseases, Düzce University Faculty of Medicine, Düzce Turkey; 3 Department of Biostatistics, Düzce University Faculty of Medicine, Düzce Turkey; 4 Department of Microbiology Reference Laboratories, National Arboviruses andViral Zoonoses Unit Public Health Institution of Turkey, Ankara Turkey

**Keywords:** Hantaviruses, neutrophil/lymphocyte ratio (NLR), platelet/lymphocyte ratio (PLR), monocyte/lymphocyte ratio (PLR), predictive factors, Turkey

## Abstract

**Background/aim:**

Hantavirus is a rodent borne zoonosis caused by the members of the virus family Bunyaviridae, genus *Hantavirus* . In this study, we aimed to determine the role of peripheral blood leukocyte ratio in differential diagnosis of Hantavirus disease.

**Materials and methods:**

The medical records of patients at the Düzce University Medical Faculty were examined retrospectively. A total of 20 patients diagnosed with hantavirus infection confirmed by serologic tests were included in the study (Group 1). The other group consisted of 30 patients suspected of hantavirus infection but found negative (Group 2). Demographic, clinical and laboratory characteristics, neutrophil/lymphocyte ratio (NLR), platelet/lymphocyte ratio (PLR), and lymphocyte/monocyte (LMR) ratios of both groups were compared.

**Results:**

As a result of the istatistics analysis, no difference was found between the groups’ age, sex, and clinical complaints except lethargy-weakness (P = 0.004) and diarrhea (P < 0.001). Hemogram analysis showed a significant difference between the groups in terms of leukocyte, hemoglobin, hematocrit, platelet, mean platelet volume (P < 0.05) and PLR (P = 0.001) and LMR (P = 0.003) values from peripheral blood leukocyte ratios.

**Conclusion:**

In conclusion, NLR, PLR, and LMR ratios may be useful for clinicians in differential diagnosis of Hantavirus in patients presenting with similar symptoms of Hantavirus disease.

## 1. Introduction

Hantaviruses, a member of the family Bunyaviridae, cause two known zoonotic infections: hemorrhagic fever with renal syndrome (HFRS) and hantavirus cardiopulmonary syndrome (HCPS) [1]. The hantavirus reservoirs in nature consist of various rodents and insectivores. In rodents, the infection is frequently asymptomatic or progresses in the form of chronic carriage. Transmission to humans occurs through respiration of aerosols contaminated with the urine, saliva, and pulmonary secretions of infected rodents [2]. HCPS leads to manifestations of diffuse pulmonary edema, impaired pulmonary functions, and cardiovascular failure, and mortality is high. This syndrome frequently occurs with the Sin Nombre, Andes, Laguna Negra and New York serotypes is widely reported on the American continent and attracted particular attention with an outbreak in 1993 [3,4]. HFRS is a disease type that generally occurs in Europe and Asia and deriving from the Dobrova, Puumala, Hantaan, Saaremaa, and Seoul serotypes. Depending on the virus serotype, the disease may range from mild to severe form. The Puumala serotype has quite low mortality [5,6]. Hantavirus was first identified in Turkey in the Black Sea region in 2009 in patients presenting with fever, abdominal pain, diarrhea, thrombocytopenia, and renal insufficiency. Hantavirus cases were subsequently reported from various Black Sea region provinces [7–9]. The principal laboratory findings in these cases, generally seen in the form of HFRS, are thrombocytopenia and creatinine elevation. Similar to other critical illnesses, identifying early and novel biomarkers and combining clinical features with laboratory parameters for predicting diagnosis and prognosis of hantavirus will be of great assistance to clinicians in terms of initiating effective treatment and improving success rates.

Peripheral blood leucocyte ratios are an important parameter that has begun being used in patients with infectious diseases in recent years. Several studies have revealed that these are useful in evaluating the etiology, course, and prognosis of such diseases [10,11]. These include the neutrophil-to-lymphocyte ration (NLR), the platelet-to-lymphocyte ratio (PLR), and the lymphocyte-to-monocyte ratio (LMR). This study compared the demographic data, clinical features, and laboratory values of patients presenting from Düzce and the surrounding area to our hospital in the Western Black Sea region in 2012–2018 and with confirmed or suspected hantavirus infection. This is also the first study to evaluate peripheral blood leucocyte ratios in the context of predicting diagnosis of hantavirus infection.

## 2. Materials and methods

### 2. 1. Study design

The medical records of patients under follow-up and treatment with a preliminary diagnosis of hantavirus infection at the Düzce University Medical Faculty, Turkey, in 2012-2018 were examined retrospectively. Twenty patients diagnosed with hantavirus infection confirmed by serologic tests were included in the study (Group 1). The other group consisted of 30 patients suspected of hantavirus infection but found negative (Group 2). This group consisted of patients who were considered as suspected cases due to the initial complaints and similar regions. Group 2 patients were discharged with different diagnosis from nonspecific infection (respiratory system, gastrointestinal tract infection etc.) or noninfection during the follow-up period. Group 2 was composed of patients who had negative serological tests both in the initial application and in the serum samples taken on the following days.

The demographic and clinical characteristics and laboratory parameters obtained at time of initial presentation of 20 patients with positive laboratory hantavirus diagnosis and 30 patients with negative findings following preliminary diagnosis were recorded. Patients’ demographic characteristics, age, sex, occupation, length of incubation, length of hospital stay, clinical findings, blood values (white blood cell [WBC], platelet count [PLT], hematocrit [Hct], hemoglobin [Hb], mean platelet volume [MPV], aspartate aminotransferase [AST], alanine aminotransferase [ALT], total bilirubin, lactate dehydrogenase [LDH], alkaline phosphatase [ALP], creatine phosphokinase [CPK], C-reactive protein [CRP], creatinine [Cr], prothrombin time [PT], activated partial thromboplastin time [aPTT], and international normalized ratio [INR]) were recorded onto forms. NLR, PLR, and LMR were calculated as the ratio of neutrophils to lymphocytes, platelets to lymphocytes, and monocytes to lymphocytes, respectively. These parameters were compared between the groups 1 and 2. 

### 2.2. Laboratory methods

Complete blood count parameters were measured by an automated hematology analyzer (Abbott Cell-Dyn Ruby; IL 60064 USA). CRP was measured from blood samples using turbidimetry (Uni Cel RDxC 800; Beckman Coulter, Pasadena, CA, USA).

For diagnosis of hantavirus infection, patients’ serum samples were sent to the National Arboviruses and Viral Zoonoses Laboratory, Microbiology Reference Laboratories and Biological Products Department of the Turkish Public Health General Directorate. The indirect immunofluorescence test (Hantavirus Mosaic 1, IIFT, immunoglobulin G [IgG] and IgM, Euroimmun, Perkin Elmer, USA) was used for serological diagnosis. The presence of antihantavirus antibodies against HTNV, Sin Nombre, PUUV, DOBV, SEOV, and Saaremaa viruses were assessed in line with the manufacturer’s instructions with 100-fold dilution. All positive samples were retested with Euroline Hantavirus Profile 1 Immunoblot testing (Euroimmun, Perkin Elmer, USA) in order to differentiate between different serotypes.

### 2.3. Statistical analysis

Distribution of data was examined using the Shapiro–Wilk test. The independent samples t test was used to compare normally distributed data between the groups, while the Mann-Whitney U test was used for data with nonnormal distribution. Pearson’s chi-square, Fisher’s Exact or Fisher-Freeman-Halton tests were used to analyze categorical data according to expected count and number of groups compared. Cut-off values for discriminating hantavirus positivity were calculated using receiver operating characteristic (ROC) curve analysis for statistically significant variables at univariate analyses. Statistical analyses were performed on SPSS v.22 software (IBM Corp., Armonk, NY, USA), and P values less than 0.05 were considered significant.

## 3. Results

### 3.1. Demographic data and clinical presentation

Mean ages were 38.6 ± 12 years in Group 1 and 45.2 ± 20 in Group 2. Men represented 95% (n: 19) of patients in Group 1 and 76.7% (n: 23) of Group 2. There was no significant difference between the groups in terms of sex or age (P > 0.05). Seventeen of the 20 patients followed-up with hantavirus positivity lived in the region of Düzce (13 in the district of Yığılca), while the other three lived in neighboring provinces. Originating from the Yığılca area of Düzce was identified as a risk factor for hantavirus positivity (P = 0.047). Apart from one patient who died from acute respiratory syndrome and cardiac arrest without renal involvement, the other patients with hantavirus infections were followed-up with a diagnosis of HFRS, and five patients (25%) received hemodialysis. Times between onset of symptoms and hospitalization were not significantly different between the groups 1 and 2, but the length of hospitalization was significantly greater in Group 1 (P = 0.003). Comparison of clinical symptoms at time of initial presentation revealed significant differences between the groups in terms of lethargy-malaise (P = 0.004) and diarrhea-abdominal pain (P < 0.001). There was no significant difference in other symptoms. The most common serotype among patients with hantavirus infection was Puumala (n: 18). Hantavirus serotype of one patient who died and five patients who received dialysis treatment were Puumala. All suspected cases of hantavirus infection were also screened for acute leptospirosis and CCHF virus using molecular and serological tests and were determined to be negative for both agents. The findings are shown in Table 1.

**Table 1:** Demographic and clinic characteristics of the two groups.

Demographic data			
Age (years)	38.6 ± 12.1	45.2 ± 20.2	0.155
Sex (male/female)	19/1	23/7	0.123
Place of residenceDüzce-Yığılca (n)Düzce-otherOther	13 (65%) 4 (20%) 3 (15%)	12 (40%)16 (53.3%)2 (6.7%)	0.047
Time between onset of symptoms and hospitalization(days) mean (min-max)	5 (1-14)	3.5 (0-10)	0.116
Length of hospitalization (days) mean (min-max)	11.5 (2-23)	6 (0-23)	0.003
Hemodialysis	5 (25%)	-	
Ex	1 (5%)	-	
Clinical findingsFeverLethargy-weaknessNausea-vomitingLumbar/back painOliguria/anuriaBurning/itching in the eyesShock	15 (75%)15 (75%)12 (60%)13 (65%)1 (5%)3 (15%)1 (5%)	17 (56.7%)10 (33.3%)15 (50%)2 (6.7%)0 (0%)2 (6.7%)0 (0%)	0.1860.0040.4870.0010.4000.6360,400
Hantavirus serotypePUUVUndifferentiated	182	--	

PUUV: Puumala virus.

### 3.2. Laboratory results

Comparison of blood tests at time of presentation among patients in the groups 1 and 2 revealed significant differences in terms of the complete blood count parameters WBC, Hb, Hct, PLT, and MPV values, and the biochemical parameters Cr, LDH, and ALP values (P < 0.05). CPK, hepatic enzymes, total bilirubin, CRP, PT, aPTT, and INR values were similar between the two groups (P > 0.05). Mean PLR and LMR values were significantly lower in patients with hantavirus infection (P = 0.001, and P = 0.003, respectively). In contrast, NLR was of no diagnostic value in predicting hantavirus infection (P = 0.289). A statistical analysis of all parameters is shown in Table 2. 

**Table 2:** Laboratory values of the two groups.

	Group 1(Hantavirus confirmed cases)n = 20	Group 2(Hantavirus unconfirmed cases)n = 30	P-value
Hematological parameters			
WBC (µL-1) mean (min-max) (n = 3–15)	12.520 (2100-29.400)	6,900 (900–16,700)	<0.001
Hgb (g/dL) (n = 8-17)	14.4 ± 2.4	11.7±2.4	<0.001
Hct (n = 26–50)	42.5 ± 6.9	34.8 ± 6.8	<0.001
Platelet count (µL-1) (min–max) (n = 50-500)	63,500 (10,400–195,000)	131,500 (8000–479,000)	0.002
MPV (n = 9-13)	9.9 ± 1.5	8.7 ± 1.1	0.001
NLR	4.3 (0.5–10.9)	3.1 (0.2–26.4)	0.289
PLR	29.6 (5.9–140.9)	109 (7.3–575)	<0.001
LMR	1.4 (0.7–118.6)	3.4 (0.7–21.5)	0.003
PT (s) (n = 11-15)	13.3 ± 3	12.4 ± 1.4	0.233
aPTT (s) (n = 21-35)	29.4 ± 8.7	27.2 ± 4.0	0.311
INR (n = 0.8–1.2)	1.1 ± 0.1	1.1 ± 0.1	0.560
Biochemical parameters			
Urea (mg/dL) (n = 17–43)	89 (14.4–291)	37.7 (12.0–293.1)	0.067
Creatinine (mg/dL) (n = 0.7–1.2)	3.4 (0.7–10.2)	1.2 (0.3–13.4)	0.006
CPK (U/L) (n = 0–171)	234.5 (19–2590)	126 (18–2360)	0.471
LDH (U/L) (n = 135–225)	473.5 ± 175.8	313.9 ± 136.9	0.001
AST (U/L) (n = 0–50)	59 (10–174.3)	38.5 (9–203)	0.488
ALT (U/L) (n = 0–41)	28 (6.4–133)	25.5 (2.5–166.7)	0.692
Bilirubin (mg/dL) (n = 0.3–1.2)	0.4 (0.2–12)	0.5 (0.1–18.6)	0.329
ALP (U/L) (n = 30–120)	59 (30–93)	80.5 (39–150)	0.001
Albumin (g/dL) (n = 3.5–5.2)	3.2 ± 0.5	3.5 ± 0.8	0.165
CRP (mg/dL) (n = 0–5)	7.3 (0.8–16.3)	5.3 (0.1–38.6)	0.699

**Figure 1:**
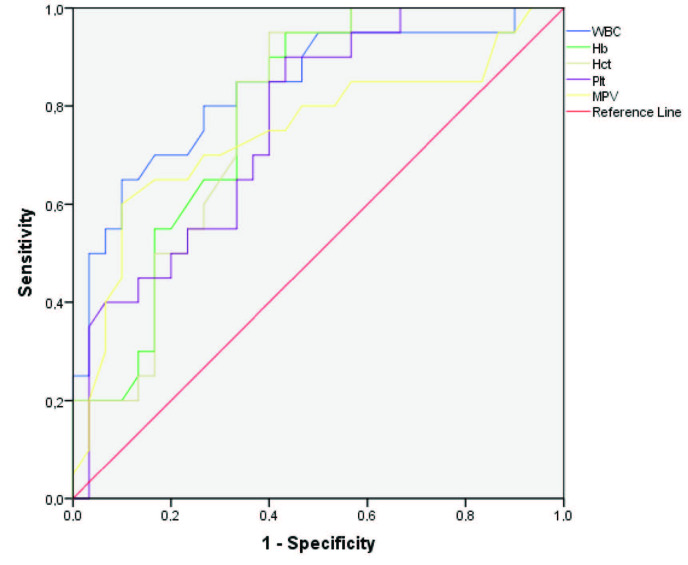
ROC curves for prediction of hantavirus positivity by WBC, Hb, Hct, Plt, and MPV

**Figure 2:**
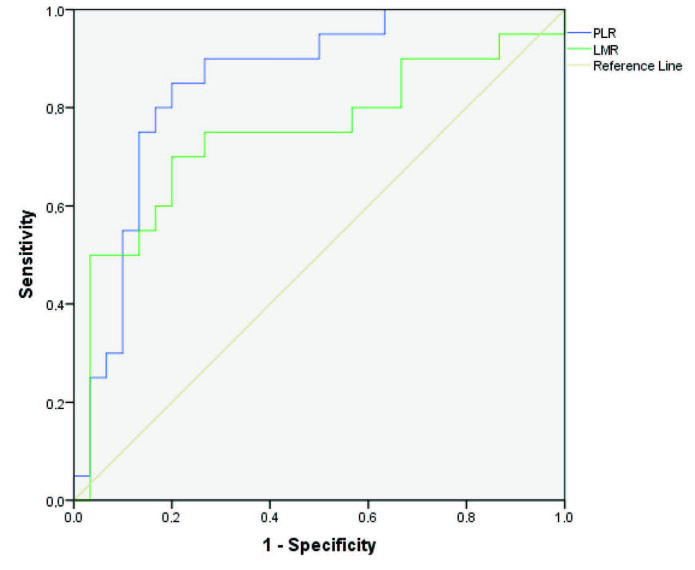
ROC curves for prediction of hantavirus positivity by PLR and LMR.

**Figure 3:**
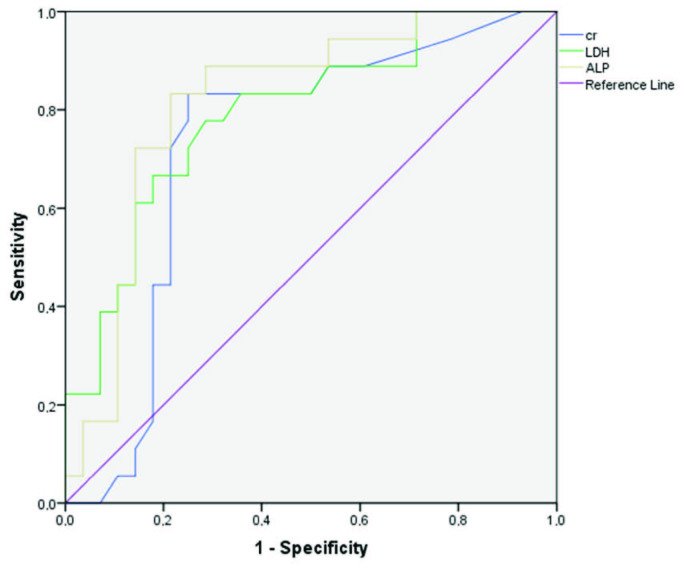
ROC curves for prediction of hantavirus positivity by cr, LDH, and ALP.

ROC analysis with AUC measurement was used to explore the predictive value of the laboratory parameters. This yielded AUC values of 0.836 for WBC, 0.786 for Hb, 0.778 for Hct, 0.763 for Plt, 0.747 for MPV, 0.850 for PLR, 0.747 for LMR, 0.730 for cr, 0.773 for LDH, and 0.793 for ALP (Table 3). ROC analysis of these parameters is given in three separate charts for complete blood count, biochemistry, and peripheral blood leucocyte ratios in Table 3 and the Figures 1, 2, and 3.

**Table 3:** Receiver operating characteristics (ROC) analysis for the calculation of the discriminative ability of laboratory markers.

	AUC	95%CI	P value	Cut–off	Sensitivity	Specificity
KR	0.730	0.583–0.877	0.006	≥ 1.88	80.0	76.7
LDH	0.773	0.637–0.910	0.002	≥ 369.5	73.7	71.4
ALP	0.793	0.664–0.922	0.001	≤ 66.5	78.9	76.7
WBC	0.836	0.718–0.954	< 0.001	≥ 8550	80.0	73.3
HB	0.786	0.661–0.910	0.001	≥ 12.6	85.0	66.7
HCT	0.778	0.651–0.905	0.001	≥ 37.45	85.0	66.7
PLT	0.763	0.631–0.894	0.002	≤ 84500	70.0	63.3
MPV	0.747	0.597–0.898	0.003	≥ 9.25	70.0	73.3
PLR	0.850	0.740–0.960	< 0.001	≤ 70.16	85.0	80.0
LMR	0.747	0.594–0.899	0.003	≤ 1.8	70.0	80.0

Subanalysis of patients receiving and not receiving dialysis therapy in the hantavirus-positive group revealed a longer length of hospitalization in subjects undergoing dialysis (P = 0.033). The subanalysis results revealed no statistically significant difference in laboratory parameters or NLR, PLR, and LMR values between the dialysis and nondialysis (P > 0.05). 

## 4. Discussion

Hantavirus affects tens of thousands of people in Asia and Europe, and has been reported from different regions of Turkey in the form of sporadic outbreaks since 2009. The majority of reported cases originate from the Black Sea region, a heavily forested area in the north of the country [7-9,12]. The Düzce region lies in northwest Turkey, largely between the 41st and 42nd north latitudes. The fact that most of our patients came from the area of Yığılca was statistically significant for hantavirus positivity. In addition to the geographical nature of Yığılca, this may also be due to homes being within the forest zone, inadequate socioeconomical conditions, insufficient hygiene conditions, and an ongoing hantavirus epidemic in the area.

Initial findings of Hantavirus infection may be confused with clinical and laboratory findings of other zoonotic infections. In a recently published article, some laboratory parameters were compared and a scoring system was established to differentiate between hantavirus and leptospirosis [13]. We performed a similar study using Hemogram parameters in patients with a prediagnosis of Hantavirus. Clinical and laboratory data at time of presentation to hospital were recorded for hantavirus-positive patients and for patients identified as hantavirus-negative following admission with a preliminary diagnosis of hantavirus infection. Differences between the two groups in terms of demographic, clinical, and laboratory parameters were then examined. We also analyzed differences between the hantavirus-positive and –negative groups in terms of the peripheral blood leucocyte ratios NLR, PLR and LMR, described as being predictive of systemic inflammation in several studies. 

Demographic characteristics of the hantavirus-positive patients in this study (male 95%, mean age 38.6 years) were consistent with Black Sea region outbreaks (male 87.5%, mean age 45.9 years) [14] as well as with outbreaks in other European countries [15]. Although hantavirus infection is independent of age and sex, the higher prevalence in males may be attributed to greater participation in outdoor activities. The most common clinical symptoms in our cases (fever, malaise, nausea-vomiting, and back pain) were consistent with previous reports from Germany, Bulgaria, and Slovenia [16-18]. Since 90% of our hantavirus-positive cases involved the Puumala serotype, no symptoms of shock or hematuria were determined. The Puumala serotype was also the agent identified in a neighboring region in 2009 [7]. Significant differences were observed between groups 1 and 2 in terms of lethargy and flank pain but none in terms of other symptoms. Length of hospital stay of the hantavirus-positive patients in our study was similar to that in Çelebi et al.’s study [14]. Additionally, length of stay was greater in the hantavirus-positive group than in the hantavirus-negative group.

Leukocytosis and thrombocytopenia are known to occur in hantavirus infections [2]. Lysis of megakaryocytes resulting from accumulation of immune complexes associated with hantavirus and depletion of platelets due to vascular damage are responsible for the development of thrombocytopenia [19,20]. Consistent with the previous literature, leucocyte, hemoglobin and hematocrit values were higher in the hantavirus-positive group than in the hantavirus-negative group, while platelet levels were lower. Wang et al. [21] also reported a relation between platelet suppression and severity of renal injury. We determined no significant relation between low platelet values and dialysis requirements at subgroup analysis. 

The kidney is the most severely affected organ in HFRS. Tubular obstruction in the kidney, ischemic damage, and immunological developing due to immune complex accumulation occur together with direct hantavirus-associated injury in the kidneys [22]. As anticipated, we therefore determined significantly higher serum creatinine levels compared to the hantavirus-negative group. Although no difference was determined between the groups in terms of hepatic enzymes, LDH values were higher, while ALP values were lower in the hantavirus-positive group. Due et al. [23] described AST levels as a prognostic factor in predicting HFRS, while Zhenjun Yu et al. [20] reported that LDH elevation was significant in discriminating mild from severe HFRS. In their study of hantavirus-positive and –negative patients, Kaya et al. [24] determined no difference in ALT and values compared to the control group; however, AST and LDH values were higher in hantavirus-positive patients. Consistent with our research, that study also observed no difference between the two groups in terms of coagulation parameters. 

Peripheral blood leucocyte ratios (NLR, PLR and LMR) are novel, inexpensive, suitable for routine use, and reproducible markers of the systemic inflammatory response. They can be simply calculated from white blood cell assay and determined under simple laboratory conditions. The numbers of studies assessing the efficacy of NLR, PLR, and LMR in the diagnosis and prognosis of various infectious diseases has continued to increase in recent years [25-28]. We encountered no previously published studies related to these parameters in hantavirus disease. 

PLR and LMR values were lower in our hantavirus-positive patient group, while NLR values were similar between the two groups. Several studies have investigated these parameters in viral or bacterial infections. One cohort study of Crimean Congo Hemorrhagic Fever patients in Turkey reported that a low PLR value was associated with blood transfusion requirements and mortality [25]. However, we determined no relation between hantavirus-positive patients’ dialysis requirements and NLR, PLR, or LMR. This may be due to our low patient number, and to the fact that our cases involved the Puumala serotype with its milder course. One study reported that low LMR was of prognostic significance in confirming influenza virus and severity of influenza in patients with influenza-like illness [29]. Another study compared patients with Bell’s palsy (BP), caused by the Herpes simplex virus, and a control group, and reported high NLR values associated with poor improvement in the BP [30]. The association between NLR and outcomes of diabetic foot infection was examined in a cohort of 75 patients. Higher NLR was observed in patients who went on to develop osteomyelitis (mean 12.3 vs 6.0, P = 0.004) [31].

**Limitations**

There are a number of limitations to our study. First, the cohort was small and the study was a single-center one. Second, advanced analysis could not be performed since no cut-off values could be calculated for laboratory parameters in dialysis comparisons in hantavirus-positive patients. Moreover, since the majority of our cases were with Puumala serotype, our results may not be capable of generalization to other serotypes. Further prospective, extensive, randomized controlled studies will therefore strengthen the present findings. 

In conclusion, hantavirus infection continues to be reported in Turkey, particularly in the north of the country. Since the serotype involved in our region and surrounding areas is generally Puumala, subclinical or milder infections are generally involved. Variations in creatinine, LDH, and platelet values in subjects presenting with hantavirus-like symptoms, together with NLR, PLR, and LMR (peripheral blood leucocyte ratios) will be of assistance to physicians in the early diagnosis of hantavirus.

## Ethical Approval

This study is in compliance with ethical standards. The study was approved by the Ethics Committee of Medical Faculty at Düzce University (approval no: 2019/104) in Düzce.
